# Supplementary material for: Are carbon emissions trading and green financial instruments synergistic? -Comprehensive quantitative research based on content analysis
Source: PLoS One. 2024 Mar 7;19(3):e0298601. doi: 10.1371/journal.pone.0298601 (PMC10919601; doi:10.1371/journal.pone.0298601)
Supplement: S1 Data — (ZIP) [file pone.0298601.s001.zip › Data/地级市Z_F工作报告.zip!/╜¡╬≈/─╧▓2╩╨/└┤╫╘╣1⁄2╓┌║┼í╛┬φ┐╦╩2╛▌═°í┐_2011.docx]

各位代表：

现在，我代表南昌市人民政府，向大会作政府工作报告，请予审议，并请各位市政协委员和列席会议的同志提出意见。

一、“十一五”经济社会发展主要成就及2010年工作简要回顾

（一）“十一五”经济社会发展主要成就

“十一五”时期，是南昌改革开放和现代化建设取得巨大成就的五年。面对国际环境的巨大变化、金融危机的巨大冲击和历史罕见的特大冰雪灾害、特大洪涝灾害等巨大挑战，在省委、省政府和市委的正确领导下，全市上下坚持以科学发展观为统领，深入贯彻省委、省政府提出的做大做强南昌和“六个走在全省前列”的要求，积极实施大开放主战略、新型工业化核心战略、新型城镇化基础战略和创业富民、创新发展战略，加快建设“一个基地、三个中心、四个南昌”，经济社会发展取得了阶段性历史成就，现代区域经济中心城市和现代文明花园英雄城市初步建成，走出了一条具有南昌特色的“打基础、优结构、蓄势能、添后劲、惠民生”的品质型发展路子。

1、经济总量五年翻番，经济质量明显优化，现代区域经济中心城市活力彰显

“十一五”期间，南昌地区生产总值年均增速14.4%以上，高于全国和全省同期年均增速4.1和1.4个百分点，2010年突破2200亿元大关，实现了五年翻番。财政总收入年均增速20.6%，2010年突破300亿元大关，实现了四年翻番。社会消费品零售总额年均增速19.5%，实现了四年翻番。规模以上工业增加值年均增速21%，实现了三年翻番。全社会固定资产投资年均增速30%以上，居中部省会城市前列，2010年接近2000亿元，实现了三年翻番。累计批准外资企业916家，实际利用外资71.05亿美元，是“十五”期间的2.62倍；海关出口总额2010年突破30亿美元大关，实现了五年翻两番。三大产业协调发展，比重调整为 5.6∶56.1∶38.3。税收收入占财政收入比重2010年达到93.1%，比2005年提高4.3个百分点，财政收入占地区生产总值比重2010年超过15.3%，比2005年提高2.8个百分点，均明显高于全国省会城市平均水平。高新技术产业增加值占全市工业增加值的比重2010年达到24%。先进制造业重要基地建设步伐加快，成为国家低碳试点城市和“十城千辆”、“十城万盏”、服务外包示范城市，建成半导体照明等4大国家级产业基地，汽车及零部件、光伏光电、航空及零部件、服务外包、生物和新医药等五大战略性支柱产业取得突破性进展，朝着千亿产业目标迈进。引进世界500强企业39家，国内200强企业37家。区域性金融、商贸、物流中心建设步伐加快，第三产业经营业态不断优化，引进沃尔玛、麦德龙、欧尚等一批世界知名商贸巨头，公共物流区建设取得新突破，物流功能加快完善；渣打银行等外资银行、股份制银行进驻南昌。非公经济快速发展，活力日益彰显。坚持以城带乡、以工促农，加大“三农”投入，农业综合生产能力进一步增强，粮食生产连续五年丰收，蔬菜、生猪、水产等主要农产品生产能力稳步提高，一批农业示范区、特色种养基地、农业产业化龙头企业蓬勃发展，农村富余劳动力加快转移，现代农业发展水平大幅提升。国有企业改革取得决定性胜利，完成或基本完成425家国有企业改制。政府机构改革顺利完成，行政效率大幅提高。国土资源管理体制改革加快推进，市本级招拍挂公开出让土地3.9万亩，是“十五”期间的3.7倍。“十一五”期间，我市入选世界十大动感都会，跻身近五年全球经济增长最快城市行列，成为60年来 32个大城市科学发展代表之一。

2、城市规模不断拓展，城市功能日益完善，现代文明花园英雄城市魅力提升

“十一五”期间，南昌建成区面积扩大到265平方公里，“一核五片、多中心、网络状组团发展”的现代化大都市发展格局初步形成。坚持规划先行，现代动感新城的承载功能明显提升。通过立法储备扬子洲土地，城市轨道交通全面开工建设，生米大桥、英雄大桥和东西外环竣工通车，南外环规划实施，南昌西客站建设加快，昌北国际机场改扩建即将竣工，“三环十一射”的城市道路骨架逐步拉开。坚持建管并重，山水绿色都城的特色魅力不断彰显。大力实施环境保护立法、城市绿化美化亮化、“八湖两河” 水系活化、城镇污水处理、农村垃圾无害化处理等工程；以村庄整治、农村社区建设、产业发展为主要内容，建设新农村村点3606个；深入推进造林绿化“一大四小”工程和“森林城乡、花园南昌”建设，森林覆盖率达到21.86%，提高了5.81个百分点，空气质量优良率连续五年居中部省会城市之首、全国省会城市前列，营造了一流的空气、一流的水质、一流的环境。坚持人文日新，历史文化名城的人文传承日趋丰厚。深入开展全国文明城市创建活动，市民文明素质和城市文明程度不断提高，“大气开放、诚信图强”的城市精神深入人心。完成八一起义纪念馆、小平小道、天香园二期、八大山人梅湖景区（一期）工程等一批重大文化设施建设，编制滕王阁景区扩建工程规划，不断唱响南昌国际军乐节等特色品牌，城市发展软实力不断增强。“十一五”期间，我市被评为国家卫生城市、国家园林城市、全国科技进步先进城市、全国人防先进城市，以“鄱湖亲水美”被评为中国十大最美丽城市之一，荣获全国双拥模范城“六连冠”。

3、民生工程持续推进，社会事业全面发展，平安和谐南昌基础牢固

“十一五”期间，累计投入民生工程资金超过109.6亿元。率先在全国实行城镇居民基本医疗保险，率先在全国实行廉租住房实物配租和租赁补贴制度，率先在全省开展失地农民纳入社保试点，率先在全省启动“光明·微笑”工程，率先在全省实行城乡九年制义务教育全免费，率先在全省实现城乡医疗救助、基本医疗保险和定点医疗机构三方网上同步结算，率先在全省实行扶贫开发重点村道路村村硬化，率先在全省完善自然灾害应急体系。人民群众共享改革发展成果，幸福感不断增强。2010年城镇居民年人均可支配收入和农民年人均纯收入分别比“十五”期末增加7975元和3251元。坚持实施“科教兴市、文化强市”战略，科技创新能力持续加强，科技对经济贡献率比“十五”期末提高10个百分点，成为国家创新型试点城市和国家知识产权示范城市。全市安排教育经费115亿元，有效化解农村学校债务问题，新建中小学校31所，全国“三大”职教中心地位进一步巩固，南昌人才进出比从“十五”期末的1∶2.3提升为“十一五”期末的2.7∶1。成功举办了第18届中国金鸡百花电影节、首届世界低碳大会等具有广泛影响力的大型文化会展活动。坚持依法治市，强化社会治安综合治理和各项安全稳定工作，群众公共安全感指数在中部省会城市中名列前茅。加强政府立法、法制建设、执法监督、廉洁自律，减少行政审批、抓好作风转变、提高行政效能，南昌发展环境进一步优化。

（二）2010年工作简要回顾

2010年，在省委、省政府和市委的正确领导下，全市上下大力实施鄱阳湖生态经济区建设和“山江湖”综合开发战略，协调推进新型工业化、新型城镇化和农业现代化进程，经济社会继续保持又好又快的发展势头。全年地区生产总值2207亿元，增速达14%；实现财政总收入322.31亿元，增长29.0%；地方财政一般预算收入146.46亿元，增长26.4%；50万元以上固定资产投资1936亿元，增长32%；社会消费品零售总额754.9亿元，增长19.5%；城镇居民年人均可支配收入18276元，增长10.9%；农民年人均纯收入7130元，增长13.2%；人口自然增长率7.8‰；全面完成了市十三届人大五次会议确定的各项目标任务。

1、三大产业增长提速、质量提升

工业经济快速上行。全市规模以上工业增加值650.92亿元，增长21.0%。55个重点企业实现增加值279.87亿元，增长23.8%。7个省级以上园区实现主营业务收入1880亿元，增长26.8%，占全市规模以上工业主营业务收入的70.1%。江铃股份30万辆整车等项目正式启动，江铜10万吨铜板带一期、宝迪3万吨肉制品深加工等项目即将投产，南昌航空工业城获得国家高技术产业基地授牌，赛维BEST高效太阳能电池、海立年产600万台空调压缩机等项目竣工投产，江铃集团成为首家销售收入超200亿元的企业集团。现代服务业加快发展。江西前湖迎宾馆等商贸物流“四个十”项目已开工开业35个，完成投资93亿元。南昌保税物流中心封关运行、南昌出口加工区（B区）通过国家验收。启动城区15个菜市场升级改造。实施“万村千乡”、“家电下乡”工程，建设改造“农家店”600家，配送中心3个。北京银行正式落户，8家证券机构和3家保险机构在昌设点，新增15家担保公司和3家小额贷款公司；组建南昌大丰村镇银行。取得中央转贷地方政府债券10亿元，发行企业债券27亿元；泰豪软件等企业加快上市步伐，上市公司实现再融资34.75亿元。引进昊威集团、东软集团等国内外知名服务外包企业，江西慧谷—用友南昌产业园正式开工，全年新增服务外包企业130家，新增从业人数2.1万人；服务外包接包合同执行金额6.57亿美元（含在岸），增长83.1%，“中国服务外包示范城市”得到夯实。接待游客1510.1万人次，增长22%；实现旅游总收入100.8亿元，增长17.4%。成功举办了第五届“中博会”、第17届中国国际广告节、第64届全国药交会、2010中国绿色食品博览会等110多项会展活动，参观人数突破500万人次，展会交易额1200亿元。农业发展基础稳固。落实强农惠农政策，发放粮食直补、农资综合补贴、农机具购置补贴4.76亿元。开展“造地增粮富民工程”，新增耕地8618亩。实施“十亿斤粮食增产”工程，全市粮食播种面积554万亩，是有统计数据以来仅次于1966年的最高年份。实现农林牧渔业总产值203.8亿元，增长5.9%。粮食总产量220.85万吨，蔬菜总产量93.91万吨，肉类总产量35.48万吨。全市新成立农民专业合作社251家，其中省级示范点15家。新增市级以上龙头企业38家、无公害农产品基地19个、无公害农产品33个、绿色食品19个、有机食品7个，建立龙头企业基地58个，带动农户91万户，户均增收3200元。非公经济增势明显。非公经济从业人员135万人，增长1.6%；预计实现增加值1230亿元，增长15.4%，占全市地区生产总值的比重为56.7%；上缴税金156.7亿元，增长30%，占全市财政总收入的比重为48.7%。

2、经济社会发展活力日益增强

各项改革顺利推进。圆满完成68家国有工业企业改革，妥善安置职工12.19万人，当期支付改制费用24.6亿元，其中发放、补交“一补三险”资金20.45亿元。非工口七大系统国企改革进展顺利，345户企业改制方案和职工安置方案通过职代会审议，安置职工5.8万人。深化行政审批制度改革，建立健全四级行政服务体系，在全省率先开通网上审批和电子监察系统。稳步推进食品药品机构改革，强化食品药品监管能力。深化公共财政体制改革，建立市区两级共同发展、财源共生、利益共享的收入增长机制和财力分配机制。加快文化体制改革，全面改革公益性文化事业单位、经营性文化单位、专业艺术表演团体的运行机制、用人机制、分配机制和投入机制。扎实推进医疗卫生体制改革，在市第九医院开展取消药品加成试点，医疗费用大幅下降。推进土地管理体制改革，全年获批建设用地5.12万亩，市本级土地出让金121.51亿元，为城市建设提供了土地和资金支持。开放型经济蓬勃发展。成功举办香港招商周、宁波（南昌）战略性支柱产业推介会、“请进来、走出去”招商活动月等重大招商活动，与英国和奥地利开展低碳合作项目试点。全市新批外资企业304家，增长109.6%；新批超千万美元项目66个，增长53.5%；实际利用外资20.14亿美元，增长27.2%；进出口总值53.04亿美元，增长52.4%，其中出口36.74亿美元，增长72.5%，连创历史新高；高新区出口创汇首次突破10亿美元,占全市的近1/3。实际利用内资509亿元，增长12.4%。

3、城镇化建设步伐不断加快

充分发挥规划龙头作用。编制完成《南昌市国民经济和社会发展第十二个五年规划纲要》和《山江湖发展战略重点布局规划》，《南昌市土地利用总体规划（2006-2020年）》获国务院正式批复，县级土地利用总体规划全部获省政府批准。编制完成一批地区控制性详规及重大交通专项规划。大力推进基础设施建设。实施新型城镇化项目271项，总投资826.94亿元。昌九城际、南昌枢纽西环线、新昌电厂一期、高新南大道等工程完工；南昌国际体育中心、湾里射击中心、轨道交通1号线、昌北机场改扩建、向莆铁路、火车站东广场改造、青山湖中北大道、昌东大道三期等重点工程加快推进。落实水利建设资金7.78亿元，完成白水湖双港电排站、实施朝阳水系综合整治。完成瑶湖大桥和麻丘互通工程建设，完成316、320国道等国省干线公路改造87公里。完成9座渡改桥，新增农村公路硬化路面1000公里，通自然村公路硬化率达53%。农田水利基本建设完成投资6亿元，修复水毁工程200余处，新增旱涝保收面积6万亩，改善灌溉面积12万亩。加大老城区改造步伐。实施打通“断头路”工程11项，沿江中北大道连通工程、洛阳路立交、艾溪湖大桥、滨江北路改线、青山湖南大道二期等项目顺利推进。实施塘子河、十字街、象山南路中段等棚户区改造工程，改善3.58万户棚户区居民居住条件。完成湖坊邓村等10个“城中村”拆迁改造面积35万平方米。全面铺开五大组团和小城镇建设。实施五大组团项目101项，完成投资30.6亿元。推进瑶湖组团基础设施建设；确定昌南组团58个基础设施项目和10个产业发展项目；九龙湖组团17条市政道路建设全部列入省重大重点调度项目；确定乐化组团5平方公里起步区建设范围；启动梅岭组团的区域交通设施和重大旅游项目建设。实施小城镇建设项目127项，完成投资18.53亿元，全面推进李渡镇等3个省级示范镇、莲塘镇等4个城关镇和向塘镇1个特色镇的小城镇建设。稳步提升城市管理水平。以迎接“中博会”为契机，打造覆盖主城区150.6平方公里的数字城管系统，实施管理重心下移，推行城市精细化、长效化管理。开展立面节点、拆迁还绿、环境卫生、交通秩序等市容环境“十大整治”，实施城区公共休闲场所和城区主出入口环境综合整治、八一大道提升改造、赣江东岸景观亮化提升改造等10项配套整治。巩固创建成果，顺利通过“国家卫生城市”复检。有序推进新农村建设。推进656个新农村村点建设，实行城乡一体化垃圾处理模式，完成88座乡镇垃圾中转站建设，在3666个村点开展农村清洁工程试点。继续改善生态环境。大力推进“森林城乡、花园南昌”建设，城郊造林41.33万亩，城区植树939.7万株。城市绿化覆盖率42.76%，绿地率40.16%，人均公园绿地8.54平方米。单位GDP能耗下降至0.84吨标准煤以下，化学需氧量控制在5.62万吨以内，二氧化硫排放总量控制在4万吨以内，全面完成节能减排目标任务。全市空气质量优良率94.2%，集中式饮用水源水质达标率100%。

4、民生及社会事业全面进步

民生工程扎实推进。投入专项资金50.1亿元，全面完成就业创业、社会保障、医疗保障等八个方面66件实事。新增城镇就业8.84万人；新增转移农村劳动力3.81万人。“4050”人员再就业人数和“零就业家庭”安置率均超额完成任务。全面构建以养老、医疗、失业、工伤、生育五项保险为核心的社会保障体系，全市“五险”参保总人数447.57万人，新增 16万人。下拨城乡救助资金4.72亿元，拨付洪涝灾害救灾资金2789.5万元，完成倒房重建356户。提高低保补差水平，城镇提高到196.5元、农村提高到81.9元。农村五保户集中供养率80%。7个县区开通城乡医疗救助、基本医疗保险和定点医疗机构三方网上同步结算，人均住院救助金额提高到6631元以上。234万农民参加新农合，参合率97.26%。解决26.18万农村人口饮水安全问题。免除义务教育阶段66万学生学杂费和教科书费，为各类家庭困难学生发放补助金3957.85万元。开展食品药品监督抽验，国家基本药物合格率100%。免费实施白内障复明手术18318例、唇腭裂患者修补手术746例，免费救治儿童先天性心脏病264例、白血病35例。落实农村部分计划生育奖励扶助对象3996人，二女不再生育家庭补充养老保险1091人，办理城乡计划生育家庭爱心保险10.5万人。儿童国家免疫规划疫苗免费接种覆盖率100%，五苗常规接种率和新增疫苗接种率90%以上。新增法律援助工作站8个，办理法律援助案件2423件。继续试行经济适用住房货币补助政策，为1500户家庭发放货币补贴9000万元；兴建廉租住房5000套、发放租赁补贴4785万元，解决2.27万户困难家庭住房问题。教育事业优先发展。编制完成《南昌市中心城区中小学布点规划》，建设6所九年一贯制义务教育学校，启动南昌一中朝阳新校区、南昌十九中红角洲新校区建设，完成抗震加固校舍10万平方米和一批农村中小学基础设施建设，城乡义务教育均衡发展水平不断提升。科技创新能力明显增强。国家医药国际创新园落户高新区，晶能光电公司国家硅基LED工程技术研究中心通过专家评审。江中药业、泰豪科技等一批企业被确定为国家科技创新型企业。高新技术产业增加值占GDP的比重为9%。“十城千辆”、“十城万盏”示范工程取得新进展，推广节能新能源汽车300辆，安装使用半导体照明灯近万盏。基层卫生服务体系不断加强。强化乡镇卫生院基本医疗设备配备，推进达标建设1个乡镇卫生院、57个村卫生室。合理调整社区卫生服务机构布局，推进社区卫生服务机构业务用房“国有化、固定化、达标化”。人才引进步伐加快。出台《关于实施“513”海外创新创业人才引进计划的若干意见》，开展中组部人才工作联系点建设和“人才管理改革试验区”调研工作，举办第三届“南昌人才活动周”系列活动，引进各类紧缺急需人才300余名。文化事业日益繁荣。八大山人纪念馆和新四军军部旧址陈列馆改扩建、江西名人雕塑园、市群众艺术馆建设进展顺利。全面完成“广播电视村村通工程”任务。举办了南昌市首届迎新春民间舞龙大赛等文化活动。精心包装天香园都市候鸟文化主题公园等16个项目，文化产业初具规模。体育事业蓬勃发展。第七届全国城市运动会各项筹备工作按时间节点有序推进。我市海模队在世界航海模型锦标赛C5级项目上实现7连冠。在第16届亚运会上，我市运动员获金牌1枚，铜牌1枚。在第十三届省运动会上，获得金牌、奖牌和团体总分三个第一。人口和计划生育工作成效明显。全市低生育水平进一步稳定，出生人口性别比持续下降，荣获全国婚育新风进万家活动示范市称号和军民共建人口计生工作先进单位。双拥工作再上台阶。率先在全省以人大立法形式完成拥军优属法规修正工作，顺利通过创建全国双拥模范城“七连冠”全省检查组检查。与此同时，大力开展国防教育、加强人防建设。新缔结国外友好城市2对，外事侨务工作成绩显著；民族宗教、对台事务等工作继续加强；司法、统计、审计、工商、物资、参事、档案、地方志、保密、老龄、妇女儿童、慈善、残疾人、社区建设、消费者权益保护、防震防灾、科学普及、质量技术监督等工作，都取得喜人成绩。

5、和谐稳定局面继续巩固

高度重视信访工作，严格落实信访工作责任制，开展“万名干部下基层、民情夜访促和谐”活动，千方百计关注民生，及时化解矛盾。加强平安南昌建设，积极开展社会治安专项整治和“天网”工程建设，构建社会治安动态防控体系。认真抓好生产安全、交通安全、消防安全、森林防火安全、产品质量安全、食品药品安全和应急管理工作，提高妥善处置自然灾害、公共突发事件的能力，御锦城社区等5个社区被评为“全国防灾减灾示范社区”。战胜了百年不遇的特大洪涝灾害，实现了“江河无倒堤、水库无垮坝、城市无内涝、人民群众无伤亡”的巨大胜利。认真做好“世博会”、 “亚运会” 、“中博会”期间的社会稳定工作，全市没有发生大规模群体性事件、重大社会治安案件、重特大火灾事故和安全生产事故，保障了人民群众生命财产安全。

6、经济社会发展环境明显优化

推进法治南昌创建，深入开展“五五”普法教育，切实提高公民的法律意识。坚持依法行政，打造法治政府。扎实推进政府立法工作，提请市人大常委会审议地方性法规3件，制定政府规章 3件，出台市政府规范性文件12件，认真清理现行政府规章和规范性文件。审结行政复议案件 82件，维护了公民、法人和其他组织的合法权益。认真做好政府信息公开工作，公开各类政府信息5.31万条。扎实开展“创业服务年”和“创先争优”活动，不断提升行政效能和服务水平。强化人大代表建议和政协提案办理工作，660件人大代表建议和政协提案全部办结，自觉接受人大及其常委会的依法监督、政协的民主监督和群众监督、舆论监督。严格落实党风廉政建设责任制，严肃查办违纪违法案件，坚决纠正损害群众利益的突出问题，加大效能监察力度，认真开展工程建设领域的专项治理，不断提高政府的执行力、决策力和公信力，营造科学发展、持续发展的良好环境。我市连续第七年被评为中国大陆最佳商业城市、台商投资“极力推荐城市”、最具投资潜力城市。

各位代表，南昌经济社会发展取得的成绩，是全市上下认真贯彻落实中央的大政方针，省委、省政府和市委的战略决策，团结拼搏、开拓创新的结果；是市人大依法监督和市政协民主监督的结果；是各民主党派、工商联、无党派人士、各人民团体和驻昌单位、人民解放军、武警官兵以及离退休老同志大力支持的结果，是全市广大干部群众共同努力的结果。在此，我谨代表市人民政府，向全市人民，向所有关心、支持南昌发展的同志们、朋友们，表示衷心的感谢并致以崇高的敬意！

我们也清醒地看到，与省委、省政府期望南昌“在六个方面走在全省前列”、成为“江西崛起的脊梁”的要求相比，与突飞猛进的发展形势相比，我们还有不小的差距，主要表现在：经济总量还不够大，综合实力还不够强，属于欠发达省会城市的基本市情没有根本改变；处于“不进则退、慢进也是退、稍有松懈就会掉队”的竞争格局中的地位没有根本改变；产业聚集水平和生产要素配置能力还不高、经济转型发展的任务依然艰巨；经济运行中的结构性矛盾和深层次问题尚未解决，影响发展的体制机制障碍还有待突破；有核心竞争力的产业和企业还不多，培育新的财政增长点和经济增长极还任重道远；城市建设融资能力、承载功能和管理水平有待加强，节约资源和保护环境任务还很重；保障和改善民生压力不小，政府效能仍需进一步提高。对此，我们将采取切实措施，认真加以解决。

二、“十二五”时期的基本构想、发展目标和主要任务

“十二五”时期，是我市坚定不移地推进“更大更强更优更美”的“两篇文章”建设黄金时期，更是我们促进南昌科学发展、进位赶超、绿色崛起的关键时期。当前，我市已经迈上人均地区生产总值4万元即6000美元的新台阶。纵观国内外主要城市的发展历程，这一阶段的突出特征是：产业结构由低层次向高层次发展，由劳动密集型向资本密集型、技术密集型转型，消费层次由低端向高端升级。站在这一新的历史平台，机遇与挑战并存，希望和困难同在。一方面，国际经济向环太平洋国家转移、沿海地区向中西部地区转移，尤其是国家把转变经济发展方式作为科学发展的主线，省委、省政府推动以南昌为核心的鄱阳湖生态经济区建设，为南昌经济社会的跨越发展提供了广阔的发展空间；另一方面，国际贸易保护主义不断抬头，发达国家控制着资本和资源的定价权，为我们产业升级和核心竞争力提升造成了不小的困难；加上国家加强土地、资金宏观调控，城市间竞争日趋激烈，对我们的工作都提出了更高要求。面对新的发展形势，站在新的历史平台，南昌要实现科学发展、进位赶超、绿色崛起，就必须以更加前瞻的视野，更加清晰的思路，更加有力的举措，更加务实的作风，不断开创经济社会又好又快发展的新局面。

（一）基本构想

根据市委对“十二五”发展的部署，今后五年我市经济社会发展的基本构想是：在新的起点上朝着“更大更强更优更美”的方向，全面建设更高水平的“两篇文章”：一是要坚持发展是第一要务，使我市经济发展速度高于全省平均水平、高于中部地区省会城市平均水平，在经济总量上再造一个新南昌；二是要按照转变经济发展方式的要求，不断优化经济结构，进一步增强城市的集聚力、辐射力和承载力，在更大范围、更高层次上引领区域经济发展；三是要加快改造提升老城区、繁荣发展新城区，加快城乡一体化进程，进一步唱响历史文化名城、山水绿色都城、现代动感新城的品牌，使城市更富活力、更具魅力、更有亲和力；四是要加快社会事业发展，大力改善民生，促进社会和谐，建设文明城市，提高百姓的幸福指数。

（二）发展目标

综合考虑未来发展的趋势和条件，今后五年全市经济社会发展的主要目标是：

第一，经济实力大幅提升。到2015年全市地区生产总值达到4500亿元，年均增长13%，人均达到12700美元；地方财政一般预算收入突破300亿元，比2010年翻一番，年均增长16%以上；在全国省会城市中的排位继续前移。

第二，经济结构更加优化。农业现代化水平显著提升，汽车及零部件、光伏光电、航空及零部件、服务外包、生物和新医药五大战略性支柱产业继续做大做强，服务业占GDP的比重年均提高1个百分点，城乡区域发展的协调性增强，经济增长的科技含量显著提高。

第三，改革开放不断深化。统筹城乡发展的行政管理体制、市场经济体制、社会管理体制等重点领域和关键环节的改革取得新突破，科学发展的体制机制不断完善。对外开放的广度和深度不断拓展，基本形成多层次对外开放格局。

第四，城市品位显著提高。城市建设力度持续加大，城市基础设施更加优化，城市功能不断完善，城市环境明显改善，城市承载力、辐射带动力和区域首位度明显提高，城镇化率年均提高1.15个百分点。

第五，生态文明不断进步。单位地区生产总值能耗和二氧化碳排放持续下降，主要污染物排放总量继续减少，能源资源利用率明显提高。“森林城乡、花园南昌”品牌进一步唱响，生态文明建设走在全国同类城市前列。

第六，社会建设明显加强。健康城市建设稳步推进，教育、科技、文化、体育和卫生事业等全面发展，城乡公共服务均等化基本实现，城乡发展更加协调，民主法治更加健全，社会管理模式加速转型，群众幸福感明显增强。

第七，人民生活显著改善。城镇居民年人均可支配收入和农民年人均纯收入较快增长，年均增长分别达到12.5%和13%，努力实现居民收入增长和经济发展同步、劳动报酬增长和劳动生产率提高同步。

（三）主要任务

1、增强投资、消费、出口牵引力，保持经济快速发展的强劲势头。保持投资持续增长，着力扩大消费需求，促进出口贸易稳定发展，形成投资、消费、出口协调拉动经济增长的良好态势。

2、坚持先进制造业和现代服务业相互推动，提升产业核心竞争力。培育壮大战略性支柱产业，做强做大先进制造业，加快发展服务业，积极发展现代农业，有序发展园区经济和产业基地，推进产业转型升级，构建现代产业体系，促进现代产业的聚集水平、辐射和竞争能力有一个大的提升。

3、加快推进新型城镇化，增强城市的影响力和辐射力。坚持新城建设与旧城改造并重，主城区发展与组团开发并重，中心城市繁荣与中小城镇发展并重，规划建设结构有序、功能完善、生态良好、特色鲜明的城镇体系，推动城市规模拓展，管理水平提升，功能丰富完善，增强在环鄱阳湖城市群中的辐射带动作用。

4、加快推进城乡一体化，力促城乡统筹发展走在全省前列。坚持以工促农、以城带乡，统筹城乡规划、产业布局、管理体制、公共服务，推进城乡一体化试点工作，促进县域经济跨越式发展，全面加快新农村建设，加速城乡一体化进程。

5、推进新一轮基础设施建设，夯实发展的支撑力和承载力。按照统筹协调、合理布局、适度超前、安全可靠的原则，重点推进交通、能源、水利、消防和信息等重大基础设施建设。完善综合交通体系，明显改善城市交通状况。加快建设清洁能源体系，加强水利设施建设，加快信息网络建设，为经济社会发展提供有力保障。

6、加强生态环境建设和环境保护，全面建设“森林城乡、花园南昌”。牢固树立绿色、环保、低碳发展理念，推进国家低碳示范城市建设、“森林城乡、花园南昌” 建设、节能减排、清洁生产和污染防治，加强土地等资源的集约节约利用与管理，大力发展循环经济，走生态文明发展之路。

7、强化公共服务和社会建设，不断提高人民群众幸福感。推进基本公共服务均等化，提高全社会就业水平，有序推进社会保障、基本养老服务、社会救助等体系建设，积极建设健康城市，抓好保障性安居工程建设，全面做好人口和计划生育工作，促进社会建设和社会组织发展，加强社会治安综合治理。

8、推进科技兴市和人才强市战略，提升发展的软实力和竞争力。加强自主创新能力建设，提升教育发展水平，建设人才高地和文化大市、文化强市，提升社会文明程度，形成南昌区域竞争新优势。

9、坚持深化改革和扩大开放，为全面做好更高水平的“两篇文章”提供制度保障和动力源泉。大力建设服务型政府和法治政府，推进重点领域和关键环节改革，充分激活全社会的积极性和创造性。不断完善对外开放软环境，大力实施“引进来”和“走出去”相结合的开放战略，增强经济社会发展的活力。

三、2011年工作安排

2011年是中国共产党建党90周年，也是“十二五”规划的开局之年，还是第七届全国城市运动会在我市举办之年。今年政府工作的总体要求是：全面贯彻落实党的十七届五中全会、中央经济工作会、省委十二届十四次全会和市委九届十一次全会精神，以科学发展、进位赶超、绿色崛起为主题，以加快转变经济发展方式为主线，以成功举办“七城会”为抓手，大力推进鄱阳湖生态经济区建设和山江湖综合开发战略，坚持先进制造业与现代服务业双轮驱动，新型城镇化与城乡一体化两翼支撑，加速推进新型工业化、新型城镇化和农业现代化进程，构建更加富民惠民和更具包容性的和谐社会，为“十二五”规划顺利实施开好头、起好步。

今年全市经济社会发展的主要预期目标是：

地区生产总值增长13%；

财政总收入增长18%；

地方一般预算收入增长18%；

全社会固定资产投资增长26%；

规模以上工业增加值增长18%；

社会消费品零售总额增长19%；

实际利用外资增长12%；

实际利用内资增长10%；

城镇居民年人均可支配收入增长12%；

农民年人均纯收入增长12.5%；

万元生产总值能源消耗比上年下降2.8%；

人口自然增长率控制在8‰以内；

居民消费价格总水平控制在4%左右。

为实现今年工作总体要求和主要预期目标，我们将采取以下主要工作举措：

（一）以办好“七城会”为抓手，统筹各项筹备工作，举全市之力把“七城会”办成“精彩难忘、绿色生态、安全和谐、隆重节俭”的体育盛会

强化组织领导。各级各部门领导要增强对“七城会”重要性的认识，主动参与“七城会”的筹备工作，形成全面参与、密切配合、相互支持的协调运行机制，坚持做到人力、物力、精力和财力向“七城会”倾斜，日常的各项工作为“七城会”服务和配套。“七城会”指挥部专职人员要集中办公、认真履职，切实把上级精神贯彻好、把筹备工作推进好、把各项任务完成好。

加快场馆及路网建设。高标准完成南昌国际体育中心、湾里射击中心建设，协调推进省奥体中心和省瑶湖水上运动中心建设。加快现有场馆改造和新闻中心、各场馆新闻发布厅及新建场馆配套路网建设。全面实施“数字城运”工程，建成“七城会”综合信息服务系统。认真实施“迎城运百路大会战”工程，营造良好外部环境。完善水电气等基础设施，提升供给能力和水平。

全力做好竞赛工作。做好赛前准备、赛风赛纪和反兴奋剂检查等工作，科学安排比赛项目，确保运动员在公开、公平、公正的前提下参与竞争、展示才能、创造佳绩，为伦敦奥运会选拔优秀人才。全力做好我市参赛团队备战工作，力争比赛成绩超历史。

精心组织重大活动。认真组织实施 “七城会”火炬传递、开幕式大型群体文艺表演、闭幕式大型国际军乐表演等重大活动及“倒计时”系列活动，充分体现“红色英雄城、绿色七城会”的特色。

营造浓厚氛围。加大官方网站、省市及中央各类新闻媒体宣传报道力度，形成强大的宣传声势。大力开展“迎城运、强素质”十大文明行动，引导全市人民以主人翁的姿态，自觉遵守法律法规、爱护基础设施、维护城市形象，自觉当好城市的讲解员、推销员、服务员。通过发行“七城会”即开型体育彩票、吸引企业赞助及特许经营等方式，积极有效推动资源开发。

做好接待服务。根据分级对口接待的原则，制定科学合理的接待方案；坚持精心组织、精心施工、精心管理，确保“七城会”运动员村如期竣工投入使用；有序开展志愿者选拔培训，提供热情周到的服务。

强化安全保障。制定安保工作方案，实施安保技防工程，落实安保队伍，健全安保机制，进行安保模拟演练，消除安保盲点，确保火炬传递、开闭幕式等重大活动、各项赛事及重点设施、重要场所的安全。抓好交通组织、食品供应、气象保障、医疗卫生等保障工作，制订应急总体预案和专项预案，做到万无一失。

（二）以转变经济发展方式为主线，协调推进三大产业，全力提升经济发展质量和水平

强力推动新型工业化。加快运用高新技术和先进适用技术改造提升传统产业步伐，促进冶金和新材料、纺织服装、家电、机电制造、食品等五大传统优势产业转型升级。大力发展战略性新兴产业，重点培育汽车及零部件、光伏光电、航空及零部件、服务外包、生物和新医药等五大战略性支柱产业。着力抓好重大项目的实施，推进江铃股份30万辆整车、南昌航空工业城、南柴K型发动机、格特拉克DCT变速箱、海立年产1200万台空调压缩机、双汇食品等项目建设，促进南昌印钞厂扩建、亚洲啤酒异地搬迁扩建、晶能光电200亿粒芯片、光伏700兆瓦硅片和1000兆瓦模组、雨润食品等项目早日竣工投产。打造特色鲜明、优势明显、具有核心竞争力的工业园区，争取新增2家省级生态工业园区试点单位，做大桑海生物医药产业基地规模和实力。实施中小企业成长工程，着力解决中小企业融资难问题，进一步减轻企业负担。严格落实节能降耗责任制，确保全年单位GDP能耗下降2.8%、规模以上工业企业单位增加值能耗下降3%。

加快发展现代服务业。做优传统商贸业，提升完善2个市级商业中心和6个区级商业中心，抓好万达商业广场、恒茂梦时代广场、家乐福、香格里拉、喜来登等重点项目建设，打造一批商业街、社区商业，推进“家电下乡”、“万村千乡”、“双百市场”、“农超对接”工程和农村现代流通服务体系建设。做强现代物流业，着力建设现代物流信息平台，加快南昌华南城、中部汽贸城大型物流业项目建设，打造昌北、昌南和昌西南3个物流基地和航空工业城码头。做大金融服务业，继续吸引银行等金融机构落户，实现小额贷款公司县（区）全覆盖，新建1家村镇银行，将南昌新世纪创投公司改组为政府引导基金公司，推进3家公司上市融资。做实服务外包业，推进东软生命健康产业科技园、用友软件产业园等项目建设；加快 “云计算”和物联网产业发展，打造完善信息技术公共配套平台，确保“服务外包示范城市”保牌进位。大力发展会展经济，办好第二届世界低碳大会、第四届中国绿色食品博览会等一批全国知名展会。繁荣发展旅游业，加快滕王阁景区扩建、梅岭景区、象湖景区、天香园三期、樟树林文化生活公园等旅游项目建设，唱响 “农家乐”品牌，完善旅游公共服务设施，强化南昌红色旅游珍品、文化旅游名品、生态旅游精品、休闲旅游新品等四大品牌推介，着力把南昌打造成全国著名的旅游集散地和目的地。

大力推进农业现代化。切实抓好“十亿斤优质粮食增产工程”、“十万吨油脂增产工程”和“菜篮子工程”建设，加快高标准农田、优质水稻基地建设，稳定粮食生产。加快推进蔬菜、生猪、家禽等八大主导产业基地和南昌县国家级，进贤县、安义县省级现代农业示范区建设。全力推进农业产业化，重点培植年产40万吨米业集团等一批省级以上龙头企业，大力扶持80个市级以上龙头企业，全市龙头企业销售收入突破400亿元。大力开展“农田水利基本建设年”活动，加快实施圩堤加固、病险水库除险加固、中小河流治理等水利工程建设。深入开展农民负担专项治理，依法推进土地承包和流转，切实维护农民根本利益。

（三）以创新体制机制为突破口，加大改革开放力度，不断增强科学发展的内在动力和活力

促进重点领域改革新突破。全面完成非工口七大系统国企改革，有序剥离国有企业承担的社会职能，促进企业健康发展。启动城市建设体制改革，提高城市建设专业化水平。深入推进土地管理体制改革，创新土地经营理念，推广“朝阳新城开发模式”，提升土地经营效益和水平。积极探索和谐拆迁新模式，努力破解城市拆迁难题。推进投融资机制改革，做大做强市城投公司、昌工控股等投融资平台。开展城乡建设用地增减挂钩试点和城乡一体化试点，积极探索农村产权制度、户籍制度改革。稳步推进国有林场垦殖场改革、事业单位及其绩效工资改革、文化体制和医疗卫生体制改革，在93家乡镇卫生院以及政府设立的社区卫生中心实施国家基本药物制度，实行公立医疗机构药品省级网上集中招标采购。进一步改善非公经济发展环境，切实解决融资难、准入难、人才紧缺、社会服务不健全等问题，进一步促进多种经济成分竞相发展。认真推进鄱阳湖生态经济区先行先试区工作。

推动经济社会发展大开放。巩固“中博会”、宁波推介会等重大招商活动成果，抓好项目跟踪推进。组建五大战略性支柱产业招商团，积极参加香港活动周、赣台会等国家、省级招商引资活动，大力开展小分队招商、驻点招商、专题招商，着力引进一批产业新、链条长、投资大、有影响的大项目以及符合产业政策、科技含量高，有利于生态环保、改善民生的好项目。加大信贷、海关、质检、外汇服务力度，重点扶持以光伏为龙头的高新技术产品开拓海外市场，促进外贸出口提速增效。充分发挥海铁联运、保税物流中心和出口加工区功能，做优对外开放平台。积极参与长珠闽区域性经济合作，促进生产要素优化配置。充分发挥省会城市和鄱阳湖生态经济区核心城市的辐射作用，着力打造“南昌经济圈”。

（四）以新型城镇化建设为核心，坚持规建管并重，全力打造宜业宜居的“森林城乡、花园南昌”

全面推进城乡建设。发挥规划龙头作用，认真执行土地利用总体规划，继续完善历史文化名城、城市交通、城镇消防等专项规划，督促指导县区认真修编各类规划。坚决拆除各类违法违章建筑，维护城市规划的权威性和严肃性。抓好重大重点工程建设，加速推进向莆铁路、昌北国际机场扩建、南昌综合运输枢纽、火车站东广场改造等重大工程建设；全面实施轨道交通1号线工程、开展2号线可行性研究；实施昌东大道三期、赣江北大道、青山湖中北大道、南京东路、青山路改造、昌南大道东延等重点项目建设，打通洛阳路隧道、沿江中北大道等6条“断头路”；推进青山湖西岸开发建设，对全市国省干线公路逐步实施“文明样板路”工程建设。加快“五大组团”和小城镇建设步伐，大力推进瑶湖、九龙湖、乐化、昌南、梅岭等五大组团起步区建设；以路网、廉租房、医院、学校等民生项目为重点，推进小城镇建设。坚持以城带乡，镇村联动，加快推进新农村示范村点工程，抓好650个新农村村点整治，推进70个市县两级新农村综合示范村点建设。

大力提升城市管理水平。进一步理顺区级城市管理体制，建立“一级监督、两级政府、三级管理、四级网络”管理体系，做到管理全覆盖。开展交通环境综合治理，加强交通安全宣传教育，突出治理交通违法行为，完善交通安全设施，新建一批过街人行通道，清理和建设一批停车场。实施公交优先战略，建设公交专用道，规划快速公交BRT系统，逐步解决行车难、停车难等突出问题。以市容市政、卫生保洁、文明素养等方面为主要内容，对88个社区进行全面综合整治，构建现代城市社区服务体系，提升社区建设整体水平。对一批城市道路、桥涵进行沥青摊铺、病害维修、除险加固。实施非法户外广告整治、立面节点改造、景观亮化美化等城市容貌提升工程。完善污水处理配套管网，推进工业园区污水处理厂建设。严格落实污染减排目标责任制，主要污染物化学需氧量、氨氮排放总量下降1%，二氧化硫、氮氧化物排放总量下降1.2%。推行低碳、生态、环保、高效的城乡一体化农村垃圾处理体系，实现减量化、资源化、无害化处理，美化城乡生态环境。

打响低碳生态城市名片。深入推进“森林城乡、花园南昌”建设，全面启动“国家森林城市”创建工作，完成城郊新造林面积23.31万亩，打造进出南昌的森林通道，实施4个山上造林项目、7个森林工业园区、24个森林城镇和200个森林村庄建设。加快推进城区绿化工程，力争城区绿化覆盖率42.85%，绿地率40.26%，人均公园绿地面积8.59平方米以上。以建设低碳试点城市为契机，大力倡导低碳生态的生活方式，推动可再生能源在建筑中应用，启动大型公共建筑节能改造。推进奥地利低碳技术示范区建设，打造红谷滩生态居住和服务业中心区、高新区国家级生态工业示范园区、湾里生态园林区和军山湖低碳农业生态旅游区等四大低碳经济示范区。

（五）以保障和改善民生为目的，共建共享发展成果，切实提高人民群众幸福感

以保障和改善民生为着力点，投入200亿元，实施160项民生工程，切实解决困难群众最迫切需要解决的问题，让人民群众得到更多实惠。

实施就业创业工程。进一步扩大城镇就业人数，提高城镇就业率，新增城镇就业6.99万人，安排“4050”人员就业0.69万人，“零就业家庭”安置率达100%。农村劳动力从事非农产业3.33万人。发放小额贷款2.5亿元。

实施社会保障工程。城镇职工基本养老保险参保缴费人数达到75.74万人，新型农村社会养老保险参保人数64.64万人，被征地农民养老保险参保人数2.3万人。继续落实计生家庭补充养老保险政策和爱心保险。城镇职工和农民工工伤保险参保人数分别达66.41万人和13.21万人。新建市慈爱老年公寓、市儿童福利院。

实施社会救助工程。建立城乡低保标准自然增长机制、社会救助资金稳定投入机制，进一步完善临时救助制度，适当扩大医疗救助的惠及范围和补助水平。推进农村敬老院整合建设，启动市救灾减灾指挥中心和市物资中心储备库建设。落实城镇大集体困难企业、国有农场、林场等困难企业未参保退休职工生活补助经费，城镇“三无”人员集中供养标准由每人每月300元提高至350元。继续实施法律援助。

实施教育助学工程。继续免除义务教育阶段学生学杂费、中职学校农村家庭经济困难学生、城镇低保家庭学生和涉农专业学生的学费。对中小学贫困寄宿生、普通高中和中职学校困难学生给予补助和资助。落实困难学生高校助学贷款风险补偿金和高考入学政府资助金。

实施公共卫生保障工程。扩大城镇职工基本医疗和城镇居民基本医疗保险范围，提高新农合参合率，完善新农合工作机制。加快推进公共卫生服务均等化。开展鄱阳湖南岸血防示范区建设。继续实施“光明·微笑”工程，开展儿童白血病、先天性心脏病免费救治。推进食品药品电子化监管建设，提升食品药品安全保障水平。

实施粮食安全保障工程。不断提升粮食流通产业发展水平，动态增加市级粮油储备规模，确保粮食安全。推进放心粮油进院校、进军营、进社区、进农村“四进”工程，完善配送中心建设，确保粮油供应质量。

实施文化体育工程。建设农家书屋168个，开展送戏下乡320场，放映电影下乡1.8万场，免费开放八大山人纪念馆、新四军军部旧址陈列馆。布设全民健身路径，建设一批基层群众体育健身场所，进一步丰富人民群众文体生活。

实施安居保障工程。推进廉租住房建设，新建廉租住房4758套，为1万户困难群众发放廉租住房租赁补贴。建设经济适用住房1500套、10万平方米。建设公共租赁住房3253套，其中安排政府统建1000套、大中专院校自建2253套。完成利字街、十字街、工人新村等3个棚户区以及下尧村、桃花三村、塘山村等10个“城中村”改造任务。为农村困难群众新建、重建住房提供救助，改善农村困难群众住房条件。

实施扶贫开发工程。大力推进扶贫开发重点村扶贫规划实施。建设水禽、花卉苗木等十大扶贫主导产业，巩固10个扶贫主导产业示范点。开展党员干部一对一结对帮扶，鼓励个私民营企业兴办扶贫项目，逐步提升贫困地区自我发展能力，减少贫困人口。

改善人民群众生产生活条件。实施7处千吨万人供水工程，解决8万人农村饮水安全问题。加快建设红角洲水厂一期工程，启动城北水厂一期工程建设。新建农村户用沼气池2000个，置换人工煤气用户8万户，提升改造18个集贸市场。

（六）以满足人民群众精神文化需求为前提，全面推进各项社会事业，进一步提高社会发展水平

切实推进教育均衡发展。继续推进城区九年一贯制义务教育学校建设，建成云飞路第一、第二学校。推进优质高中工程，加快建设南昌一中朝阳新校区和十九中红角洲新校区。实施示范性职业院校品牌建设工程。促进教育资源重点向农村倾斜，缩小城乡教育差距。加快农村中等职业教育免费进程，因地制宜解决农民工子女上学和农村留守儿童教育问题。加快发展学前教育，提高农村幼儿入园率。支持发展高等教育。实施城镇教育园区建设，抓好中小学危房改造及校舍安全工程。

大力推动科技进步。继续推进科技创新“六个一”工程，积极推动“十城千辆”和“十城万盏”工程，推广应用节能新能源汽车300辆，安装LED路灯8000盏。创新产学研合作机制，增强协同创新驱动力。创新科技中介服务体系建设，建设南昌科技创新中心和科技活动中心。深入实施知识产权战略，推进知识产权质押融资、科技担保、科技保险、创业投资等投融资服务。广泛开展科学普及工作，提高全民科学素质。实施人才强市战略，加快推进人才改革试验区建设。

全面启动“健康南昌”建设。改善健康设施，加强街道（乡镇）、社区（村）体育场地建设和管理，优化提升公共健身体育场所和器材，构筑15分钟体育健身圈。提升健康服务，抓好疾病预防控制，加强卫生监督执法，提高全民健康素养，提升突发公共卫生事件应急处置能力；强化中医、妇幼保健等特色医院和社区卫生服务机构建设；推进市中西医结合医院、洪都中医院异地兴建、市第三医院朝阳院区、市中心医院、市第一医院东院等项目建设；逐步构建 “以居家养老为基础、社区服务为依托、机构养老为补充”的覆盖城乡的基本养老服务体系。培养健康人群，以“人人具备基本卫生知识、人人具有基本健康理念、人人具有健康生活方式”为重点，普及健康知识，开展健康活动，关注健康心理。优化健康环境，以创建“健康社区”为抓手，巩固南昌低碳发展理念，确保主要环境指标达到健康城市要求。

加快公共文化事业发展。完成西湖市民文化广场、八大山人纪念馆改扩建工程，加快市群众艺术馆、南昌·中国雕塑艺术馆建设，启动南昌博物馆、赣江市民公园三期、市采茶剧团搬迁等项目建设。加快文化产业发展，推进泰豪国际动漫产业园、新世界广场等项目建设。举办第四届中国·南昌国际军乐节，推出一批特色鲜明、群众喜闻乐见的优秀作品。精心组织群众文化和全民阅读活动，加强文化遗产保护，积极开展“扫黄打非”专项整治行动。

加强人口和计生工作。严格人口计生目标管理，认真贯彻落实人口计生工作“一票否决”制，稳定低生育水平，加大出生人口性别比综合治理力度，完善落实利益导向政策，提高出生人口素质，促进人口长期均衡发展。

（七）以开展“发展提升年”活动为载体，提升城市文明程度，营造良好发展环境，全力构建更加包容的和谐稳定社会

争摘全国文明城市桂冠。加强市民文明素质教育，印发《南昌市民文明礼仪读本》，建设社区精神文明电子宣传栏，整治校园周边环境，打造未成年人校外活动综合素质教育基地。做好全国婚育新风进万家示范市工作，进一步巩固国家卫生城市、国家园林城市创建成果，积极争创全国无障碍建设城市。突出南昌特色，加大拥军优属、拥政爱民工作力度，夺取全国双拥模范城“七连冠”，力争摘得全国文明城市桂冠。

维护社会和谐稳定。切实做好“保供应、稳物价、保民生”工作，加强市场监管，建立物价上涨和低保标准挂钩联动机制，开展送温暖活动，保障群众基本生活。建立健全利益诉求、协调和补偿机制，畅通市长热线、市长信箱、信访维稳、315维权等渠道，加强社会矛盾排查调处。积极探索用群众工作统揽信访工作的机制体制，认真落实领导干部接访制度、包案制度和信访稳定大排查制度，切实维护人民群众利益。有效开展“六五普法”依法治理工作。树立安全发展理念，严格落实消防和安全生产工作领导责任制、事故责任追究制，认真开展专项检查和隐患整治，坚决防止重特大火灾事故和安全生产事故发生。深化平安南昌创建，继续推进“天网工程”建设，扩大视频监控覆盖面。进一步加强专职巡防员队伍建设，全面构建社会治安动态防控体系。积极开展社会治安综合整治，严厉打击各类违法犯罪活动，坚决做到治乱点、除隐患、保安全。全面推行社会稳定风险评估机制，对涉及群众切身利益的重大工程项目和重大政策，在决策前进行经济效益和社会稳定风险“双评估”。逐步推行居住证制度，加强和改进流动人口的服务管理工作。加强灾害天气监测预警预报，提高应对极端灾害天气能力。超前部署和完善各类应急预案，加强指挥和应急演练，切实提高突发公共事件的应急处置能力，保护人民群众生命财产安全，营造和谐稳定的发展环境。

面对新的发展形势和重大发展机遇，全市各级政府要进一步增强使命感、责任感和紧迫感，切实加强政府自身建设，大力提高领导科学发展的能力。一是强化法治意识。加强政府立法，完善行政监督。坚持依法行政，严格按照法定权限和程序行使权力，履行职责。规范行政执法行为，努力做到有权必有责、用权受监督、违法受追究、侵权须赔偿。推进政府信息公开工作，保障公众知情权、参与权和监督权。二是强化民主意识。坚持做到问政于民、问需于民、问计于民，广泛听取不同阶层的意见和建议，自觉接受市人大及其常委会的法律监督和市政协的民主监督，高度重视新闻舆论和社会公众的监督，支持监察、审计部门依法独立监督。认真办理人大代表建议和政协提案，巩固提高代表委员满意率。定期向市人大及其常委会报告工作，向市政协通报情况，密切与各民主党派、工商联、无党派人士和社会各界的联系。三是强化效能意识。巩固机关效能年和创业服务年成果，扎实开展“发展提升年” 活动，着力提高政府依法履职能力、高效办事能力、破解难题能力、狠抓落实能力。充分利用信息化技术和现代化办公手段，完善政府工作流程，简化项目审批手续，缩短审批时限，进一步提升机关办事效能和服务水平。对“吃拿卡要”的行为坚决查处，对损害发展环境的行为坚决打击，不断创优发展环境。四是强化服务意识。牢固树立“全心全意为人民服务”的宗旨意识，完善“民情夜访”工作的评价、督查和奖惩机制，实现规范化、制度化、标准化和长效化。把群众呼声作为第一信号，把解决群众困难作为第一责任，全力为百姓办实事、办好事。五是强化廉政意识。牢固树立“以人为本、执政为民”的理念，进一步推进惩治和预防腐败体系建设，落实党风廉政建设责任制，增强贯彻落实《廉政准则》的自觉性，加强作风建设，大力提倡艰苦奋斗，坚决制止奢侈浪费，努力创造清正、廉洁、高效、为民的政务环境。

各位代表，回首过去五年，我们豪情满怀。展望未来发展，我们信心百倍。让我们紧密团结在以胡锦涛同志为总书记的党中央周围，在省委、省政府和市委的正确领导下，紧扣“科学发展、进位赶超、绿色崛起”的主题，大力弘扬“大气开放、诚信图强”的城市精神，坚定信心、团结拼搏、创新进取、扎实苦干，全面超额完成各项目标任务，以实际行动迎接建党90周年和第七届全国城市运动会在我市胜利召开！
